# Social isolation improves the performance of rodents in a novel cognitive flexibility task

**DOI:** 10.1186/s12983-019-0339-4

**Published:** 2019-11-15

**Authors:** Xin-Yuan Fei, Sha Liu, Yan-Hong Sun, Liang Cheng

**Affiliations:** 1 School of Psychology & Key Laboratory of Adolescent Cyberpsycology and Behavior (CCNU) of Ministry of Education, Central China Normal University, Wuhan, 430079 China; 2 Fisheries Research Institute, Wuhan Academy of Agricultural Sciences, Wuhan, 430207 China

## Abstract

**Background:**

Social isolation, i.e., the deprivation of social contact, is a highly stressful circumstance that affects behavioral and functional brain development in social animals. Cognitive flexibility, one of the essential executive brain function that facilitates survival problem solving, was reported to be impaired after social isolation rearing. However, most of the previous studies have focused on the constrained aspect of flexibility and little is known about the unconstrained aspect. In the present study, the unconstrained cognitive flexibility of Kunming mice (*Mus musculus*, *Km*) reared in isolation was examined by a novel digging task. The exploratory behavior of the mice was also tested utilizing the hole-board and elevated plus maze tests to explain the differences in cognitive flexibility between the mice reared socially and in isolation.

**Results:**

The results demonstrated that the isolated mice had a higher success rate in solving the novel digging problem and showed a higher rate of exploratory behavior compared with the controls. Linear regression analysis revealed that the time it took the mice to solve the digging problem was negatively associated with exploratory behavior.

**Conclusions:**

The data suggest that social isolation rearing improves unconstrained cognitive flexibility in mice, which is probably related to an increase in their exploratory behavior. Such effects may reflect the behavioral and cognitive evolutionary adaptations of rodents to survive under complex and stressful conditions.

## Background

Social interactions play a central role in the everyday life of many organisms, and exposure to conspecifics is critically important for behavioral development during ontogeny [1, 2]. Rodents are social animals that normally live in groups and are reared together. They interact with conspecific individuals after birth or hatching. Thus, social isolation (i.e., the deprivation of social contact) during rodent adolescence is a highly stressful circumstance that affects behavior and structural and functional brain development [3–7]. Behavioral and functional brain changes in rodents by social isolation rearing have been characterized and include enhanced aggressive and anxious behavior [8–10], increased locomotor activity [11, 12], deficits in sensorimotor gating [13–15], and cognitive dysfunction [16, 17].

Cognitive flexibility, an essential executive brain function that contributes to changing the behavior of an organism depending on situational demands, is very important for rodent survival in complex and constantly changing surroundings. Cognitive flexibility may facilitate survival problem solving in rodents by altering their strategies in response to changes in external rules or internal conditions [18–20]. Previously, several studies have used rodent models to investigate the influence of social isolation on cognitive flexibility and reported behavioral impairments in reversal learning tasks after isolation rearing [21, 22]. For example, Li and colleagues [22] reported that isolation rearing impaired the reversal learning of adult rats in a rotating T-maze task. A study by Han et al. showed reversal learning impairment in rats following brief, early isolation rearing using a forced swimming task [23]. Reversal learning tasks are usually used to assess the cognitive flexibility of experimental animals. These tasks require the animals to first to learn a reward contingency and then to detect it after it has been switched to its exact opposite, that is, the response that was previously associated with a reward is now associated with a non-reward, and vice versa [24]. However, cognitive flexibility, especially in real problem solving, is not restricted to shifting between a constrained set of options (constrained cognitive flexibility). It is also involved in complex situations in which there is a broader, unconstrained set of possible solutions (unconstrained cognitive flexibility) that require a novel solution [25]. The distinct features of these two aspects of flexibility may indicate that they are distinct cognitive processes governed by distinct neural mechanisms. However, the unconstrained cognitive flexibility of rodents following social isolation rearing is still unknown. Thus, in the present study, we examined unconstrained cognitive flexibility in mice reared under social isolation with the use of a novel digging task in which the rodent must develop a novel solution in order to obtain the reward [25].

In rodents, novel solutions to a problem (task) have been reported to be related to novel exploratory behaviors, and a positive correlation was found [26]. Moreover, some other studies in rodents reported increased exploratory or investigatory behaviors following social isolation [27, 28]. We, therefore, hypothesize that isolation rearing influences the performance of rodents in the novel digging task by increasing exploratory behavior, which reflects the unconstrained cognitive flexibility of the mice.

## Methods

### Animals and grouping

Twenty Kunming mice (*Mus musculus*, *Km*; both sexes; weaning age, 21 days old) purchased from the Center for Disease Control and Prevention of the Hubei province of China were used in the study. They were randomly divided into two groups: isolation-reared group (isolates, *n* = 10) and socially reared group (socials, *n* = 10). Each isolate was housed in a single plastic cage (26 cm × 16 cm × 12 cm) with sawdust covering the bottom of the cage, whereas five socials were house together in the same type of cage. The isolates and socials were kept in the same room (isolation-reared mice were able to smell, hear, and even see the other mice) with a natural light cycle and free access to food and water. The mice were allowed to reach a body weight of at least 35 g before food restriction, which was imposed at 6 weeks postweaning to maintain the mice at 85% of their free-feed body weight. The unconstrained cognitive flexibility test (novel digging task) started 8 weeks after weaning. After this test, the mice were transferred to the hole-board apparatus and the elevated plus maze to examine their exploratory behaviors. The ambient temperature was maintained at 20–25 °C.

### Unconstrained cognitive flexibility test

**Fig. 1:**
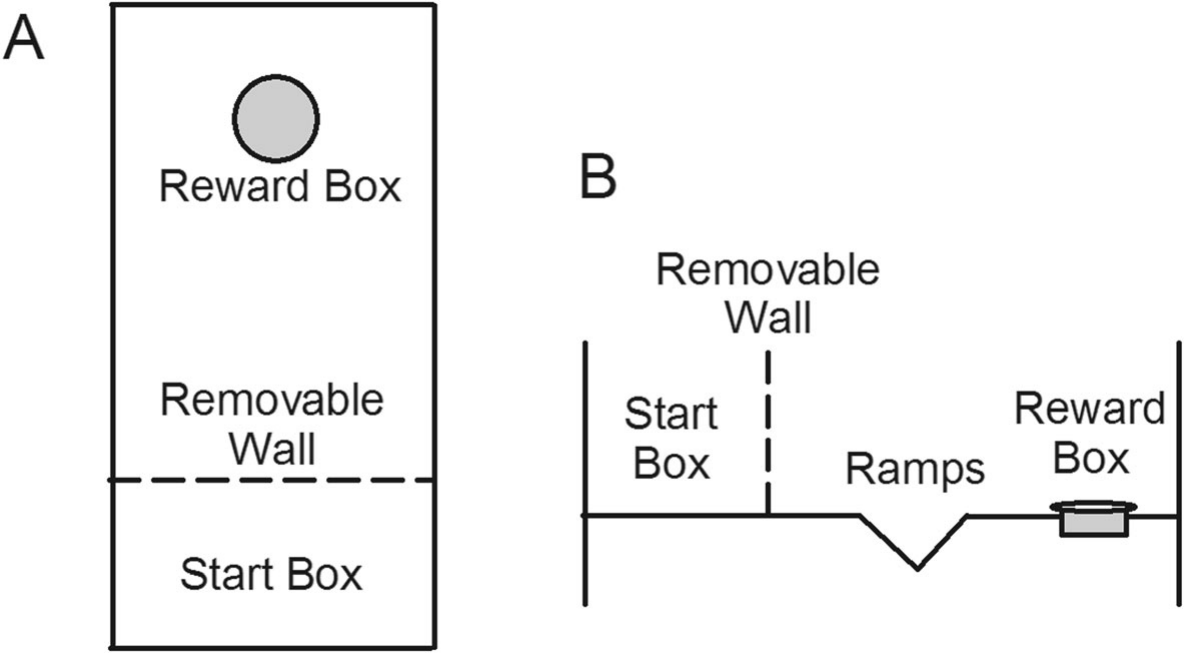
Schematic diagram of the testing apparatus for unconstrained cognitive flexibility viewed from above (**a**) and from the side (**b**). Dimensions of the apparatus was 50 cm × 10 cm × 25 cm (l × w × h). A removable plexiglas wall separated one-third of the apparatus to make a start box. When the wall was removed, the test area was exposed, consisting of a ramp leading to the reward pot at the end of the apparatus. A panel was placed in the middle of the ramp making 4 cm of open space at the bottom of the ramp. For the digging task on test day, the ramp was covered by sawdust to a height of 6 cm from the bottom to restrict access to the reward pot

The apparatus for the unconstrained cognitive flexibility test was a custom-built white Plexiglas cage modified according to Hecht et al. [25] and Thompson et al. [29], as shown in Fig. 1. The apparatus was 50 cm × 10 cm × 25 cm (l × w × h). A removable white wall separated one-third of the apparatus as a start box, in which the mouse was placed in while the apparatus was cleaned following each training or test trial. The rest area with a reward pot (5 cm in diameter, 2 cm in depth) placed at the end of the apparatus was used for the unconstrained cognitive flexibility test. When tests started by removing the wall, the test area was exposed revealing two ramps at either end that sloped downward toward the center. A Plexiglas panel was placed in the middle of the ramp allowing 4 cm of open space at the bottom of the ramp. The mice had to pass the open space of the ramps to reach the reward pot at the end of the apparatus.

The testing procedure required a total of 3 days to complete for each mouse, consisting of 2 training days and 1 testing day. Before all of the training and testing procedures, the mice were transferred to the testing room and allowed to habituate for 30 min. On training day 1, the mice were given 12 trials to retrieve the reward from the pot in the apparatus after 5 min of habituation in the start box, with guidance if necessary. During the training, the floor of the ramp in the apparatus was lightly coated with sawdust, thus permitting the mice to freely walk down the apparatus without having to dig to access the reward pot. After the training, the mice were transferred to their home cages and then back to their housing room. On training day 2, the mice were given six trials to retrieve the reward from the pot after 5 min of habituation in the start box. The floor of the ramp in the apparatus was still lightly coated with sawdust allowing the mice to freely walk down the apparatus to access the reward pot. On the testing day, instead of being lightly coated with sawdust, the ramp in the apparatus was covered by sawdust to a height of 6 cm from the bottom to block the 4 cm open space of the ramp leading to the reward pot at the end of the apparatus. The mice started from the start box and were given 30 min to finish the task of digging through the sawdust to retrieve the reward from the pot at the end of the apparatus. The time it took to retrieve the reward was marked as the completion time.

### Hole board test

The hole board test was used to examine the exploratory behavior of the mice following different raising conditions. The test apparatus comprised a plexiglas box (50 cm × 50 cm × 50 cm) with four 3 cm diameter holes that were equally spaced on the floor. For test, each mouse was placed in the center of the apparatus and allowed a free exploration for 5 min. The number of head dips (HDs) and rearing activity (RE) (the mice stood on their hind paws) and the latency to first HD were recorded as indicators of exploratory behaviors. The apparatus was cleaned with 75% alcohol after each trial.

### Elevated plus maze

The elevated plus maze was also used to test the exploratory behavior of the mice. The apparatus was a cross-shaped platform placed 50 cm above the ground, consisting of two open (30 cm × 6 cm, l × w) and two closed arms (30 cm × 6 cm × 10 cm, l × w × h) connected by a central open platform (5 cm × 5 cm). Before the test, the mice were transferred to the test room for 3 h of habituation. Then, the mice were put on the central platform heading to the open arm and were allowed 5 min to explore the apparatus. During that period, the number of HDs on the open arms and central platform and RE on the closed arms were recorded as indicators of exploratory behavior. In addition, the total number of times that the mice entered the open (OE) and closed arms (CE) and the time staying on the open (OT) and closed arms (CT) were recorded, and ΔE ((OE − CE)/(OE + CE)) and ΔT ((OT-CT)/(OT + CT)) were also calculated as indicators of exploratory behavior. The apparatus was cleaned with 75% alcohol after each trial.

### Statistical analysis

Data were analyzed and plotted with SPSS 13.0 and SigmaPlot 10.0, respectively. To examine the effects of rearing condition on the success rate of the digging task and latency to solve the digging task, Cox’s *F*-test survival analysis was conducted. A one-way ANOVA was used to examine the effects of rearing condition on exploratory behavior as determined by the hole-board test and elevated plus maze test. Furthermore, to determine whether the performance of the mice on the hole-board test and elevated plus maze test predicted the ability of the mice to solve the digging task, a regression analysis was conducted. The significance level was set at *P* < 0.05.

## Results

**Fig. 2:**
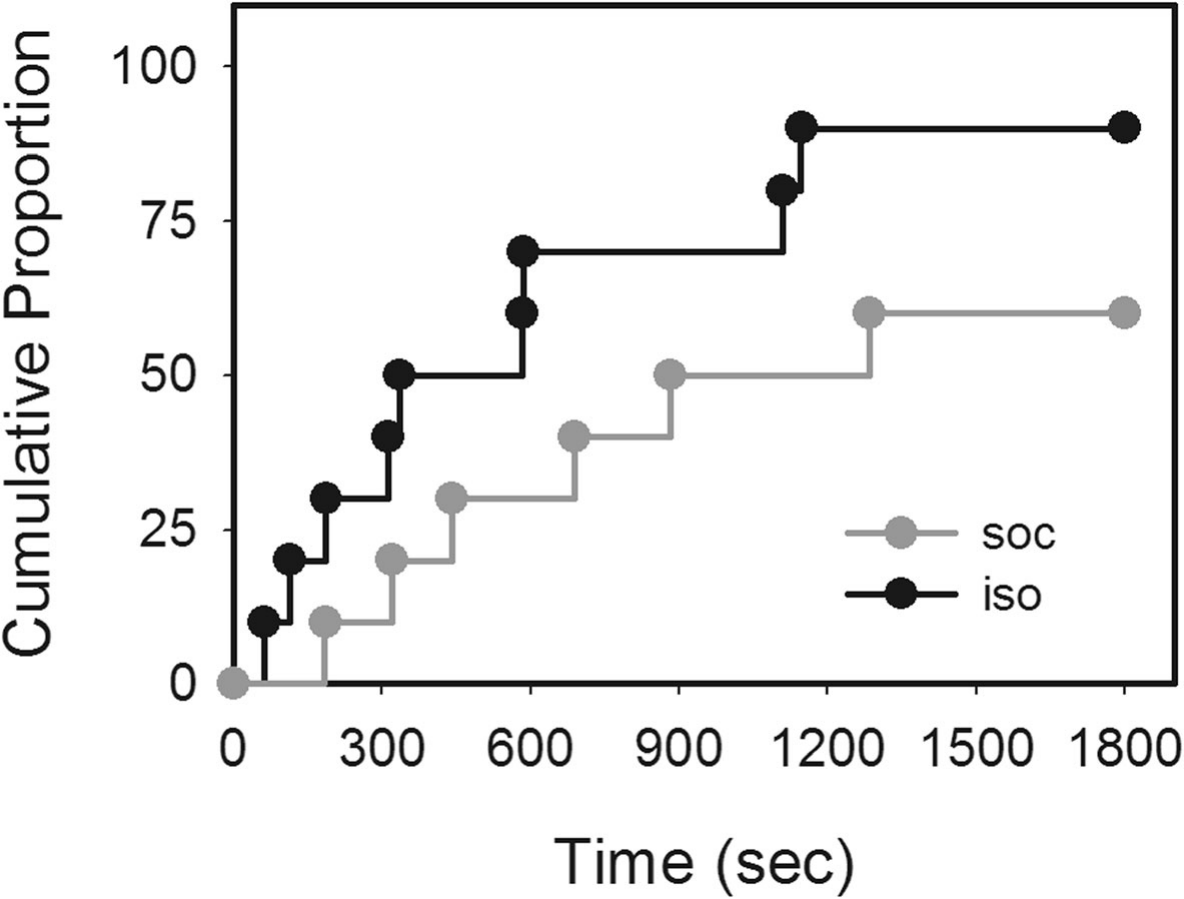
Cumulative proportion of mice reared under different conditions that solved the novel digging task. The success rate of the isolated mice (iso, black line) was significant higher than the mice reared socially (soc, gray line) (Cox’s *F*-test, *P* = 0.038)

To determine the influence of social isolation on unconstrained cognitive flexibility, the performance of the isolated mice in a novel digging task was examined and compared to that of the socially reared control mice. Results showed that isolation significantly improved the performance of the mice in the novel digging task compared with the social controls, as shown in Fig. 2. The success rate of solving the digging task of the isolated mice reached 90% while the socially reared mice had a success rate of 60% (Cox’s *F*-test, *P* = 0.038).

**Fig. 3:**
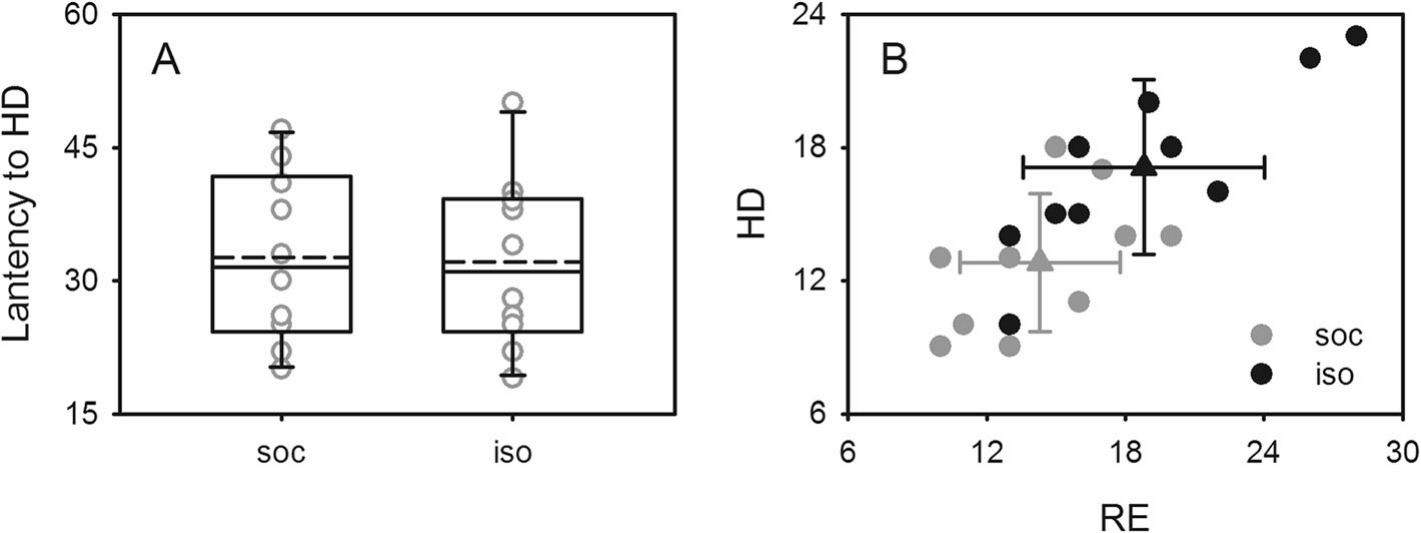
Comparison of exploratory behavior of mice reared under different conditions in the hole board test. **a**: box plots show the distribution of latency to first head dip (HD) of mice in socially reared (soc) and isolation-reared (iso) groups. Solid lines in the boxes are median lines; dashed lines in the boxes are mean lines. **b**: the distribution of rearing activity (RE) and HDs of mice in socially reared (gray circles) and isolation-reared groups (black circles). The triangles show the mean values of the RE and HDs of mice and the error bars indicate the standard deviations

**Fig. 4:**
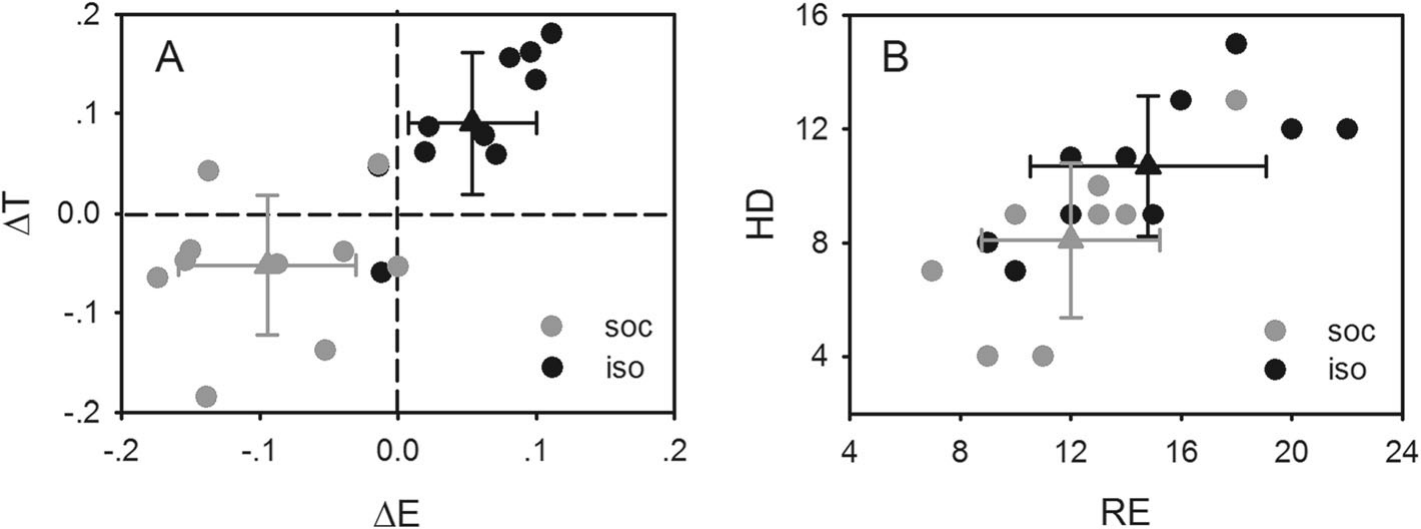
Comparison of exploratory behavior of mice reared socially (gray circles) and in isolation (black circles) in the elevated plus maze test. **a**: the distribution of ΔE and ΔT of mice in the two groups which were calculated according to the times the mice entering into open and closed arms in the elevated plus maze and the time the mice stayed there (see Methods for details). The dashed lines indicate the zero of ΔE and ΔT. **b**: the distribution of RE and HDs of mice in the two groups. The triangles show the mean values and the error bars indicate the standard deviations

**Fig. 5:**
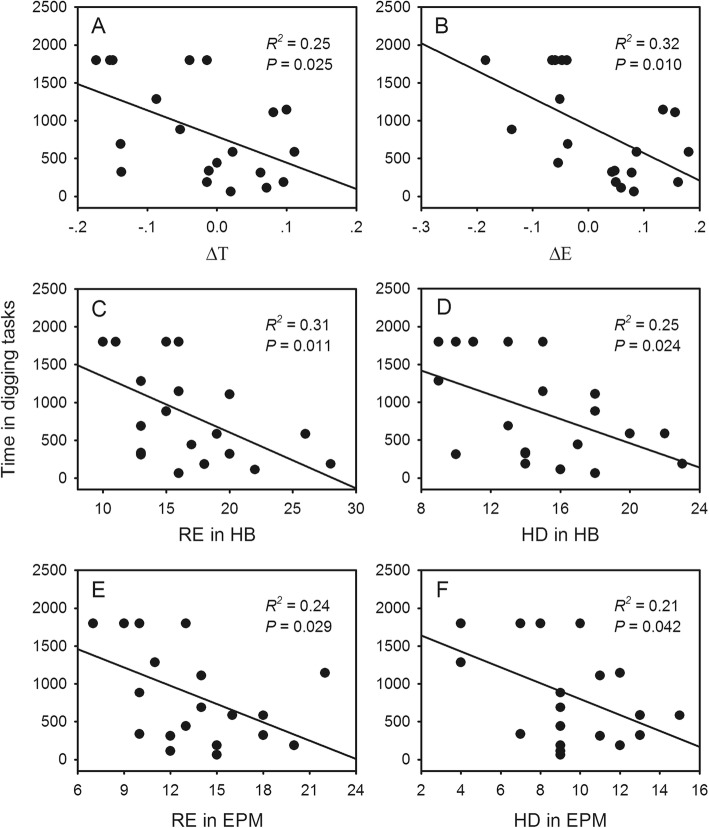
Relationship of time that mice spent in the novel digging task to the exploratory behavior level detected utilizing the hole board (HB) (**a**, **b**, **c** and **d**) and elevated plus maze (EPM) (**e** and **f**) tests. Solid lines, regression lines; *R*^*2*^, determination coefficient; *P*, significant level

To determine whether the better performance of the isolated mice in the digging task was related to changes in exploratory behavior, the exploratory behavior of the mice under different rearing conditions was examined by the hole-board (HB) and elevated plus maze (EPM) tests, and then correlations between performance in the digging task and exploratory behavior were examined. In the hole-board test, although latency to the first head dip (HD) had no significant difference between the social and isolated mice (one-way ANOVA, *F*_1,18_ = 0.014, *P* = 0.909; Fig. 3a), the total number of HDs and rearing (RE) displays of the isolated mice were significantly higher than that of the social mice (one-way ANOVA, *F*_1,18_ = 5.153, *P* = 0.036 and *F*_1,18_ = 7.347, *P* = 0.014; Fig. 3b), illustrating that social isolation increased the exploratory behavior of the mice. Similarly, the elevated plus maze test also demonstrated an increase in the exploratory behavior of the isolated mice. The values of ΔE and ΔT (see Methods for details; one-way ANOVA, *F*_1,18_ = 35.197, *P* < 0.001 and *F*_1,18_ = 20.301, *P* < 0.001; Fig. 4a) and the total number of HDs (one-way ANOVA, *F*_1,18_ = 5.028, *P* = 0.038; Fig. 4b) of the isolated mice in the elevated plus maze test were significantly greater than that of the social mice. The regression analysis revealed that the time it took the mice to complete the digging task had a negative association with exploratory behavior (liner regression analysis, *R*^*2*^ = 0.25–0.32, *P* = 0.010–0.042; Fig. 5). That is, the mice that completed the digging task the fastest showed more exploratory behavior than those that completed the task slowly or not at all. As expected, this suggests that isolation rearing influenced the unconstrained cognitive flexibility of the mice by increasing their exploratory behavior.

## Discussion

This study was designed to examine how social isolation rearing affects unconstrained cognitive flexibility in mice utilizing a novel problem-solving task, which is analogous to the tasks recently utilized to examine creative problem solving in other animals, such as the rook and the elephant [30, 31]. To accomplish this goal, the mice were randomly assigned to be reared socially or in isolation after weaning for 8 weeks. Subsequently, they were asked to complete a novel digging task, in which the mice had limited experience and were required to develop a novel solution of digging through sawdust to obtain the reward. The results demonstrated that the mice reared in isolation were faster and more successful in completing the digging task (Fig. 2), suggesting that isolation rearing positively affects unconstrained cognitive flexibility.

Social isolation is usually considered as a negative stressor that causes several kinds of behavioral and neural functional disorders in humans and animals, including enhanced aggressive behavior [10, 32, 33] and locomotor activity [11, 12, 34, 35], sensorimotor gating deficiencies [13–15, 36–38], and cognitive dysfunction [16, 17, 39, 40]. However, in the present study, greater unconstrained cognitive flexibility was found in isolated mice than in social mice, suggesting that, contrary to previous studies, social isolation could induce a positive effect on some cognition processes. To investigate the mechanisms underlying the improved unconstrained cognitive flexibility, the exploratory behavior of the mice reared socially and that of the mice reared in isolation were examined and their relationship to the performance of the mice in the novel digging task was examined. Results showed that the isolation significantly increased the exploratory behavior of the mice (Figs. 3, 4), consistent with previous findings [12, 41], and the mice with high levels of exploratory behavior were faster at solving the novel digging problem (Fig. 5). This indicated that the increase in the exploratory behavior of the mice reared in isolation was associated with or contributed to the increased unconstrained cognitive flexibility. Previously, neural physiological and pharmacological studies have shown that social isolation could decrease inhibitory control in mice [23, 42, 43]. Such inhibitory deficits may cause a decrease in novel habituation likely inducing a persistence in exploration, which was shown to benefit novel problem solving [28, 44, 45]. Novel problem solving that requires more remote potential options reflects the ability of unconstrained cognitive flexibility [25]. Thus, it is conceivable that inhibitory control deficits might be the neural basis for increases in novel exploration and subsequently for the improvement in unconstrained flexibility. Moreover, as gregarious animals, mice normally live in groups and are adapted to supporting and interacting with conspecific individuals [1–3, 46, 47]. However, isolation rearing would interrupt these relationships and individual mice would be forced to navigate their the complex surroundings and survival problems alone, which may increase investigation motivation to compensate for the absence of social support [12, 41]. That might be another potential basis to cause the exploration enhancement and the subsequent improvement of unconstrained cognitive flexibility. Certainly, it is necessary to further investigate the specific neural mechanisms that lead to increased exploration following isolation rearing.

Although previous studies have shown that social isolation leads to increased exploratory behavior, a completely opposite trend in cognitive flexibility to that reported here was found [22, 23, 41]. We postulate that this inconsistency is due to the different aspects of cognitive flexibility that were investigated in these studies and ours. Cognitive flexibility can be separated into two relatively mutually independent groups: constrained and unconstrained cognitive flexibility [24, 25]. Constrained cognitive flexibility is the set-shifting ability of cognition between a limited and constrained set of options. It is usually assessed by reversal learning tasks [22, 48]. Unconstrained cognitive flexibility requires the capacity to access more remote potential alternatives or an unconstrained set of possible solutions and is usually measured utilizing novel problem-solving tasks [25]. The distinct features of constrained and unconstrained cognitive flexibility may determine distinct neural and cognitive effects by an increase in exploratory behaviors. As mentioned above, extensive deficits in the inhibitory control of mice could be induced following social isolation [42, 43]. Such inhibitory control deficits, especially attentional inhibitory deficits, would constrain the perseveration of exploratory behavior to one set option due to the inability to effectively inhibit previously learned experiences, which is detrimental to set shifting between two constrained options [22, 49]. In addition, increases in exploration would further augment this perseveration. Thus, constrained cognitive flexibility in mice would possibly be impaired by increased exploratory behavior after isolation rearing. However, in the novel digging task that was used to model unconstrained cognitive flexibility, it was not necessary to inhibit the previously learned experiences of the mice for them to solve the novel digging problem. Therefore, the increase in exploration may directly improve the performance of the mice in the novel digging task [44, 45].

Notably, one point emerging from our results deserves further consideration. In the present study, differences in unconstrained cognitive flexibility were found between mice reared socially and in isolation. We do not interpret these findings in terms of decreases in anxiety, despite the fact that the isolated mice preferred to enter and stay on the open arm in the elevated plus maze task compared to the social mice (Fig. 4) [50–52]. Instead, our findings suggested that the exploratory demand/behavior of the mice increased considerably and that overrode any effects caused by increased anxiety. In the present study, we found that the exploratory demand/behavior of the mice increased following isolation rearing, but no significant difference was observed in latency to the first HD during the hole-board test (Fig. 3a), which conformed with the behavioral performance of mice with increased anxiety [41, 53, 54]. Moreover, previous animal studies also confirmed that social isolation could increase exploratory behavior as well as anxiety-related behavior in several behavioral tests [41, 55]. All of these reports support the conclusion that social isolation rearing could improve the unconstrained cognitive flexibility performance of mice by increasing their exploratory behavior, not by reducing anxiety. This further illustrates that the alteration of anxiety induced by social isolation was not the primary factor that influenced the unconstrained cognitive flexibility performance of the mice in this study. However, future studies are necessary to monitor various behavioral markers of anxiety to see how they relate to unconstrained cognitive flexibility performance. In addition to anxiety, other potential negative effects induced by isolation should also be monitored in future studies.

In the present study, we found that social isolation improved the unconstrained cognitive flexibility of mice and that this was related to an increase in exploratory behavior. Our finding provides an alternative viewpoint to the other studies that reported negative effects on constrained cognitive flexibility. This suggests that, even as a traditional negative stressor, social isolation can induce positive effects on cognition and behavior [21–23]. What is the neural basis of such novel findings? Neurochemistry changes in the brains of rodents reared under social isolation that parallel the behavioral effects caused by social isolation have been shown in previous studies, such as dopaminergic, serotonin, and adrenergic functional changes [4]. Future studies are necessary to examine whether these or other specific neurochemical alterations account for changes in unconstrained cognitive flexibility performance.

## Conclusion

Social isolation is usually considered a negative stressor that causes several kinds of behavioral and neural functional disorders in human and animals. However, in the present study, increased unconstrained cognitive flexibility was found in mice that were reared in isolation and this was related to an increase in exploratory behavior. Our results suggest that, contrary to only inducing negative effects, social isolation can also induce positive effects on cognition processes and behavior. Such effects may reflect the behavioral and cognitive evolutionary adaptation of rodents to survive in complex and stressful situations.

## Data Availability

The datasets used and/or analyzed during the current study are availablefrom the corresponding author on reasonable request.

## Abbreviations

CE: Entering into the closed arms
CT: Time staying in the closed arms
EPM: Elevated plus maze test
HB: Hole board test
HD: Head dip
iso: isolated
OE: Entering into the open arms
OT: Time staying in the open arms
RE: Rearing activity
soc: social

## References

[1] Arakawa H (2018). Ethological approach to social isolation effects in behavioral studies of laboratory rodents. Behav Brain Res.

[2] Meaney M, Stewart J (1981). A descriptive study of social development in the rat (Rattus norvegicus). Anim Behav.

[3] Arakawa H (2005). Interaction between isolation rearing and social development on exploratory behavior in male rats. Behav Process.

[4] Fone KCF, Porkess MV (2008). Behavioural and neurochemical effects of post-weaning social isolation in rodents—relevance to developmental neuropsychiatric disorders. Neurosci Biobehav Rev.

[5] Mirescu C, Peters JD, Gould E (2004). Early life experience alters response of adult neurogenesis to stress. Nat Neurosci.

[6] Rapoport JL, Addington AM, Frangou S, Psych MR (2005). The neurodevelopmental model of schizophrenia: update. Mol Psychiatry.

[7] Schubert MI, Porkess MV, Dashdorj N, Fone KC, Auer DP (2009). Effects of social isolation rearing on the limbic brain: a combined behavioral and magnetic resonance imaging volumetry study in rats. Neuroscience..

[8] Rodriguez-Romaguera J, Stuber GD (2018). Social isolation co-opts fear and aggression circuits. Cell..

[9] Ieraci A, Mallei A, Popoli M (2016). Social isolation stress induces anxious-depressive-like behavior and alterations of neuroplasticity-related genes in adult male mice. Neural Plast.

[10] Wei XY, Yang JY, Dong YX, Wu CF (2007). Anxiolytic-like effects of oleamide in group-housed and socially isolated mice. Prog Neuro-Psychopharmacol Biol Psychiatry.

[11] Cerbone A, Sadile AG (1994). Behavioral habituation to spatial novelty: interference and noninterference studies. Neurosci Biobehav Rev.

[12] Varty GB, Paulus MP, Braff DL, Geyer MA (2000). Environmental enrichment and isolation rearing in the rat: effects on locomotor behavior and startle response plasticity. Biol Psych.

[13] Bakshi VP, Geyer MA (1999). Ontogeny of isolation rearing-induced deficits in sensorimotor gating in rats. Physiol Behav.

[14] Fitzgerald ML, Pickel VM (2018). Adolescent isolation rearing produces a prepulse inhibition deficit correlated with expression of the NMDA GluN1 subunit in the nucleus accumbens. Brain Struct Funct.

[15] Powell SB, Swerdlow NR, Pitcher LK, Geyer MA (2002). Isolation rearing-induced deficits in prepulse inhibition and locomotor habituation are not potentiated by water deprivation. Physiol Behav.

[16] Cheng L, Wang SH, Jia N, Xie M, Liao XM (2014). Environmental stimulation influence the cognition of developing mice by inducing changes in oxidative and apoptosis status. Brain and Development.

[17] Lu L, Bao G, Chen H, Xia P, Fan X, Zhang J, Pei G, Ma L (2003). Modification of hippocampal neurogenesis and neuroplasticity by social environments. Exp Neurol.

[18] Dajani DR, Uddin LQ (2015). Demystifying cognitive flexibility: implications for clinical and developmental neuroscience. Trends Neurosci.

[19] Friedman NP, Miyake A (2017). Unity and diversity of executive functions: individual differences as a window on cognitive structure. Cortex..

[20] Izquierdo A, Brigman JL, Radke AK, Rudebeck PH, Holmes A (2017). The neural basis of reversal learning: an updated perspective. Neuroscience..

[21] Amitai N, Young JW, Higa K, Sharp RF, Geyer MA, Powell SB (2014). Isolation rearing effects on probabilistic learning and cognitive flexibility in rats. Cogn Affect Behav Neurosci.

[22] Li N, Wu X, Li L (2007). Chronic administration of clozapine alleviates reversal-learning impairment in isolation-reared rats. Behav Pharmacol.

[23] Han X, Wang W, Xue X, Shao F, Li N (2011). Brief social isolation in early adolescence affects reversal learning and forebrain BDNF expression in adult rats. Brain Res Bull.

[24] Boulougouris V, Dalley JW, Robbins TW (2007). Effects of orbitofrontal, infralimbic and prelimbic cortical lesions on serial spatial reversal learning in the rat. Behav Brain Res.

[25] Hecht PM, Will MJ, Schachtman TR, Welby LM, Beversdorf DQ (2014). Beta-adrenergic antagonist effects on a novel cognitive flexibility task in rodents. Behav Brain Res.

[26] Kaufman AB, Butt AE, Kaufman JC, Colbert-White EN (2011). Towards a neurobiology of creativity in nonhuman animals. J Comp Psychol.

[27] Sahakian BJ, Robbins TW, Iversen SD (1997). The effects of isolation rearing on exploration in the rat. Anim Learn Behav.

[28] Zimmermann A, Stauffacher M, Langhans W, Wurbel H (2001). Enrichment-dependent differences in novelty exploration in rats can be explained by habituation. Behav Brain Res.

[29] Thompson R, Huestis PW, Shea CN, Crinella FM, Yu Y (1990). Brain structures important for solving a sawdust-digging problem in the rat. Physiol Behav.

[30] Bird CD, Emery NJ (2009). Insightful problem solving and creative tool modification by captive nontool-using rooks. Proc Natl Acad Sci.

[31] Foerder P, Galloway M, Barthel T, Moore DE, Reiss D (2011). Insightful problem solving in an asian elephant. PLoS One.

[32] Wongwitdecha N, Marsden CA (1996). Social isolation increases aggressive behaviour and alters the effects of diazepam in the rat social interaction test. Behav Brain Res.

[33] Matsumoto K, Pinna G, Puia G, Guidotti A, Costa E (2005). Social isolation stress-induced aggression in mice: a model to study the pharmacology of neurosteroidogenesis. Stress..

[34] Ashby DM, Habib D, Dringenberg HC, Reynolds JN, Beninger RJ (2010). Subchronic MK-801 treatment and post-weaning social isolation in rats: differential effects on locomotor activity and hippocampal long-term potentiation. Behav Brain Res.

[35] Hickey AJ, Reynolds JN, Beninger RJ (2012). Post-weaning social isolation and subchronic NMDA glutamate receptor blockade: effects on locomotor activity and GABA signaling in the rat suggest independent mechanisms. Pharmacol Biochem Behav.

[36] Ko CY, Wang SC, Liu YP (2016). Sensorimotor gating deficits are inheritable in an isolation-rearing paradigm in rats. Behav Brain Res.

[37] Ko CY, Liu YP (2015). Isolation rearing impaired sensorimotor gating but increased pro-inflammatory cytokines and disrupted metabolic parameters in both sexes of rats. Psychoneuroendocrinology..

[38] Irvine DRF (2018). Plasticity in the auditory system. Hear Res.

[39] Cacioppo JT, Hawkley LC (2009). Perceived social isolation and cognition. Trends Cogn Sci.

[40] Manni L, Aloe L, Fiore M (2009). Changes in cognition induced by social isolation in the mouse are restored by electro-acupuncture. Physiol Behav.

[41] Thorsell A, Slawecki CJ, El Khoury A, Mathe AA, Ehlers CL (2006). The effects of social isolation on neuropeptide Y levels, exploratory and anxiety-related behaviors in rats. Pharmacol Biochem Behav.

[42] Morgan MJ, Einon DF, Nicholas D (1975). The effects of isolation rearing on behavioural inhibition in the rat. Quart J Exp Psychol.

[43] Shao F, Jin J, Meng Q, Liu M, Xie X, Lin W, Wang W (2009). Pubertal isolation alters latent inhibition and DA in nucleus accumbens of adult rats. Physiol Behav.

[44] Benson-Amram S, Holekamp KE (2012). Innovative problem solving by wild spotted hyenas. Proc Biol Sci.

[45] Borrego N, Gaines M (2016). Social carnivores outperform asocial carnivores on an innovative problem. Anim Behav.

[46] Moreno A, Gumaste A, Adams GK, Chong KK, Nguyen M, Shepard KN, Liu RC (2018). Familiarity with social sounds alters c-Fos expression in auditory cortex and interacts with estradiol in locus coeruleus. Hear Res.

[47] Schiavo JK, Froemke RC (2019). Capacities and neural mechanisms for auditory statistical learning across species. Hear Res.

[48] Hurtubise JL, Howland JG (2017). Effects of stress on behavioral flexibility in rodents. Neuroscience..

[49] Crider A (1997). Perseveration in schizophrenia. Schizophr Bull.

[50] Leo LM, Almeida-Corrêa S, Canetti CA, Amaral OB, Bozza FA, Pamplona FA (2014). Age-dependent relevance of endogenous 5-lipoxygenase derivatives in anxiety-like behavior in mice. PLoS One.

[51] Lamberty Y, Gower AJ (1996). Arm width and brightness modulation of spontaneous behaviour of two strains of mice tested in the elevated plus-maze. Physiol Behav.

[52] Polissidis A, Zelelak S, Nikita M, Alexakos P, Stasinopoulou M, Kakazanis ZI, Kostomitsopoulos N (2017). Assessing the exploratory and anxiety-related behaviors of mice. Do different caging systems affect the outcome of behavioral tests?. Physiol Behav.

[53] Takeda H, Tsuji M, Matsumiya T (1998). Changes in head-dipping behavior in the hole-board test reflect the anxiogenic and/or anxiolytic state in mice. Eur J Pharmacol.

[54] Crawley JN (1985). Exploratory behavior models of anxiety in mice. Neurosci Biobehav Rev.

[55] Koike H, Ibi D, Mizoguchi H, Nagai T, Nitta A, Takuma K, Nabeshima T, Yoneda Y, Yamada K (2009). Behavioral abnormality and pharmacologic response in social isolation-reared mice. Behav Brain Res.

